# Vagal nerve activity and cancer prognosis: a systematic review and meta-analysis

**DOI:** 10.1186/s12885-025-13956-w

**Published:** 2025-03-31

**Authors:** Wen-Bo Huang, Heng-zhou Lai, Jing Long, Qiong Ma, Xi Fu, Feng-Ming You, Chong Xiao

**Affiliations:** 1 TCM Regulating Metabolic Diseases Key Laboratory of Sichuan Province, Hospital of Chengdu University of Traditional Chinese Medicine, Chengdu, China; 2 Institute of Oncology, Chengdu University of Traditional Chinese Medicine, Chengdu, China; 3 Oncology Teaching and Research Office of Chengdu University of Traditional Chinese Medicine, Chengdu, China

**Keywords:** Heart rate variability, Vagus nerve, Cancer, Prognosis, Meta-analysis

## Abstract

**Background:**

The prognostic significance of vagal nerve (VN) activity, as measured by heart rate variability (HRV) in cancer patients remains a subject of debate. The aim of this meta-analysis was to evaluate the association between various HRV parameters and cancer prognosis.

**Methods:**

We conducted an extensive search of the PubMed, Embase, Cochrane, and Web of Science databases and compared the overall survival (OS) of cancer patients with high and low HRV. The data type was unadjusted hazard ratio (HR). Random or fixed-effects models were used to calculate the pooled HR along with the 95% Confidence Interval (CI). We used funnel plot analysis to evaluate potential publication bias.

**Results:**

A total of 11 cohort studies were included with 2539 participants. The methodological quality of the included studies is generally high. Compared with low standard deviation of normal-to-normal intervals (SDNN) group, higher SDNN was a protective factor for OS in patients with cancer (*I*^*2*^ = 66%, HR = 0.59, 95% CI: 0.46–0.75, *P* < 0.0001). Compared with low root mean square of successive differences (RMSSD) group. The prognostic value of RMSSD did not reach statistical significance (*I*^*2*^ = 0%, HR = 0.85, 95% CI: 0.70–1.03, *P* = 0.11). Among the frequency domain indicators, higher high-frequency power HRV (HF-HRV) and low-frequency power HRV (LF-HRV) were associated with significantly longer overall survival compared to the low HF-HRV and LF-HRV groups (*I*^*2*^ = 6%, HR = 0.59, 95% CI: 0.43–0.80, *P* = 0.006 and *I*^*2*^ = 74%, HR = 0.45, 95% CI: 0.22–0.93, *P* = 0.03). In the nonlinear indicators, higher maximal diagonal line length (Lmax), mean diagonal line length (Lmean), percent of recurrence (REC), and determinism (DET) were associated with poorer tumor OS. The funnel plot shows that there is no publication bias in the study.

**Conclusions:**

The findings of this study demonstrate that HRV parameters, particularly SDNN, HF-HRV, and nonlinear indices, exhibit predictive value for prognosis in cancer. Furthermore, it can be inferred that elevated VN activity may predict prolonged survival outcomes. However, these findings should be interpreted with caution due to the heterogeneity observed across included studies. Future research should prioritize prospective studies with standardized measurement protocols to validate these associations.

**Supplementary Information:**

The online version contains supplementary material available at 10.1186/s12885-025-13956-w.

## Introduction

The vagus nerve (VN) is a major component of the parasympathetic nervous system and the most widely distributed cranial nerve, regulating multiple systems including the cardiovascular, respiratory, and digestive systems [1–3]. Several cohort studies in humans and experimental studies in animals support that an intact VN may have a protective effect in various cancer. For example, vagotomy increases the risk of colorectal cancer [4–6], gastric cancer [7, 8], prostate cancer [9], breast cancer [10, 11] and pancreatic tumorigenesis [12]. Furthermore, VN stimulation may also inhibit tumor metastasis or occurrence [13–15] and regulate immune function and [16, 17].

Heart rate variability (HRV) refers to the variation in consecutive heartbeats and is primarily influenced by the cardiac VN, it is considered an indirect and non-invasive method for assessing vagal tone [18]. HRV metrics can be calculated using three different methods: time-domain analysis, frequency-domain analysis, and nonlinear dynamic analysis [19]. The time domain parameter includes standard deviation of normal-to-normal intervals (SDNN), root mean square of successive differences (RMSSD); The main frequency domain parameter includes high-frequency power HRV (HF-HRV) and low-frequency power HRV (LF-HRV); Nonlinear parameters include the mean diagonal line length (Lmean), maximal diagonal line length (Lmax), percent of recurrence (REC), determinism (DET) and Shannon entropy (ShanEn) [20].

HRV has been identified as a predictor of adverse outcomes in patients with conditions such as myocardial infarction [21], congestive heart failure [22], and renal failure [23]. However, it’s predictive value regarding survival outcomes in cancer patients remains controversial. Although previous systematic reviews and meta-analyses has suggested that higher HRV might be associated with longer cancer survival [24, 25], subsequent studies have presented contradictory findings. McGovern et al. found that SDNN and RMSSD may have limited prognostic value for survival in patients undergoing potentially curative surgery for colorectal cancer [26]. Strous et al. also found no significant correlation between the linear parameters and overall survival (OS) [27]. Compared to traditional linear parameters, nonlinear parameters can more effectively characterize the complexity of cardiac regulatory mechanisms in cancer patients [28]. Therefore, a meta-analysis is warranted to systematically and comprehensively evaluate the prognostic value of different HRV parameters in cancer patients.

## Materials and methods

### Data sources and search strategy

This study was conducted based on the Preferred Reporting Items for Systematic Reviews and Meta-Analyses (PRISMA) guidelines and the Cochrane Collaboration [29], and followed the PICOS principle (Supplementary Table 1). This meta-analysis has been registered in the International Prospective Register of Systematic Reviews (PROSPERO)(crd.york.ac.uk/PROSPERO/display_record.php? RecordID = 568076).

A comprehensive search was conducted across the PubMed, Embase, Cochrane, and Web of Science databases using both subject headings and free-text terms (Heart rate variability, HRV, cancer, carcinoma, survival, outcome, prognosis) for a structured literature review. More details on the retrieval strategy are shown in the supplementary materials (Supplementary Table 2). The last search was conducted on August 20, 2024.

### Study selection

Inclusion criteria:

1. Cancer patients with higher and lower HRV (Both linear and nonlinear parameters) were compared in the study.
2. OS was included in the study endpoints.
3. Reported or extractable unadjusted or available hazard ratio (HR) value for the univariate analysis.

Exclusion criteria:

1. Full text was not available.
2. Patients with heart disease or related medication use were excluded from the study.
3. Case reports, review articles, editorials, and letters were excluded. If the same data were published multiple times, the most recent or highest quality publication was selected.

### Data extraction

Two authors (W.B.H. and H.Z.L) independently extracted study characteristics, methodological quality, and HR using a pre-specified form, followed by cross-checking to ensure consistency, when inconsistencies arose between the two researchers during the screening or extraction process, they first re-examined the original literature and exchanged opinions to achieve consensus. If the initial discussion did not resolve the disagreement, a third researcher (L.J) was consulted to review and arbitrate the matter, with the final decision based on this arbitration. When HR were presented as figures, the required HR values were extracted using the software Engauge Digitizer (version 12.1).

### Exposure definition

The different HRV parameters have distinct meanings and cannot be used interchangeably [30]. Therefore, we included the commonly used HRV parameters: time -domain parameter (SDNN, RMSSD), frequency -domain parameter (HF, LF) and Nonlinear parameters (Lmean, Lmax, REC, DET, ShanEn).

### Risk-of-bias assessment

The quality of the included studies was assessed by the Newcastle‒Ottawa Scale (NOS) [31], which were classified into three levels: low (7 or more), moderate (4–6), and high risk (3 or less) of bias. The final results were visualized using R software 4.1.3 (R, Foundation, Vienna, Austria).

### Preplanned subgroup analyses

To address potential heterogeneity arising from methodological and clinical variations in HRV assessment, we prespecified the following subgroup analyses based on factors known to modulate HRV metrics, SDNN < 100 ms indicates mild decrease in heart rate variability, SDNN < 50 ms indicates significant decrease in heart rate variability, and SDNN < 20 ms indicates severe abnormality [32].

1. Cancer types (respiratory system cancers, digestive system cancers and other).
2. Cut-off value of SDNN (SDNN < 20 ms, 20 ms ≤ SDNN ≤ 50 ms and SDNN > 50 ms).
3. Population (European and American, Asian).

### Statistical analysis

The results of the meta-analysis were obtained using RevMan Manager (version 5.3, The Cochrane Collaboration), with both fixed-effects and random-effects models applied. Sensitivity analysis was conducted utilizing STATA statistical software (version 18, STATA Corp). Visualization was performed using R software 4.1.3, with the following R packages for plotting: grid (version 4.2.2), forestploter (version 0.2.3), pheatmap (version 1.0.12).

A meta-analysis was conducted using HR values for OS. HR data were presented as dichotomous variables, providing a point estimate and a 95% CI for each effect size. *P* < 0.05 was considered statistically significant.

Heterogeneity was evaluated by the Chi-squared (*Chi*^*2*^) test with significance set at a p-value of 0.10, and *I*^2^ statistic was used to quantify heterogeneity. We considered *I²* < 50% as indicative of low heterogeneity, allowing for the use of a fixed-effects model in the meta-analysis. For *I²* > 50%, which suggest high heterogeneity, a random-effects model was applied. Galbraith radial plot was constructed to systematically investigate potential sources of heterogeneity.

## Sensitivity analysis

Stable results enhance credibility, whereas significant changes require caution in interpretation and drawing conclusions [33]. Therefore, in this study, a sensitivity analysis was performed by excluding each study individually.

### Analysis of publication bias

Publication bias refers to the tendency for research results to be published or not published based on the nature and direction of the findings [34]. In this study, a funnel plot was used to assess publication bias.

## Results

### Study selection

**Fig. 1:**
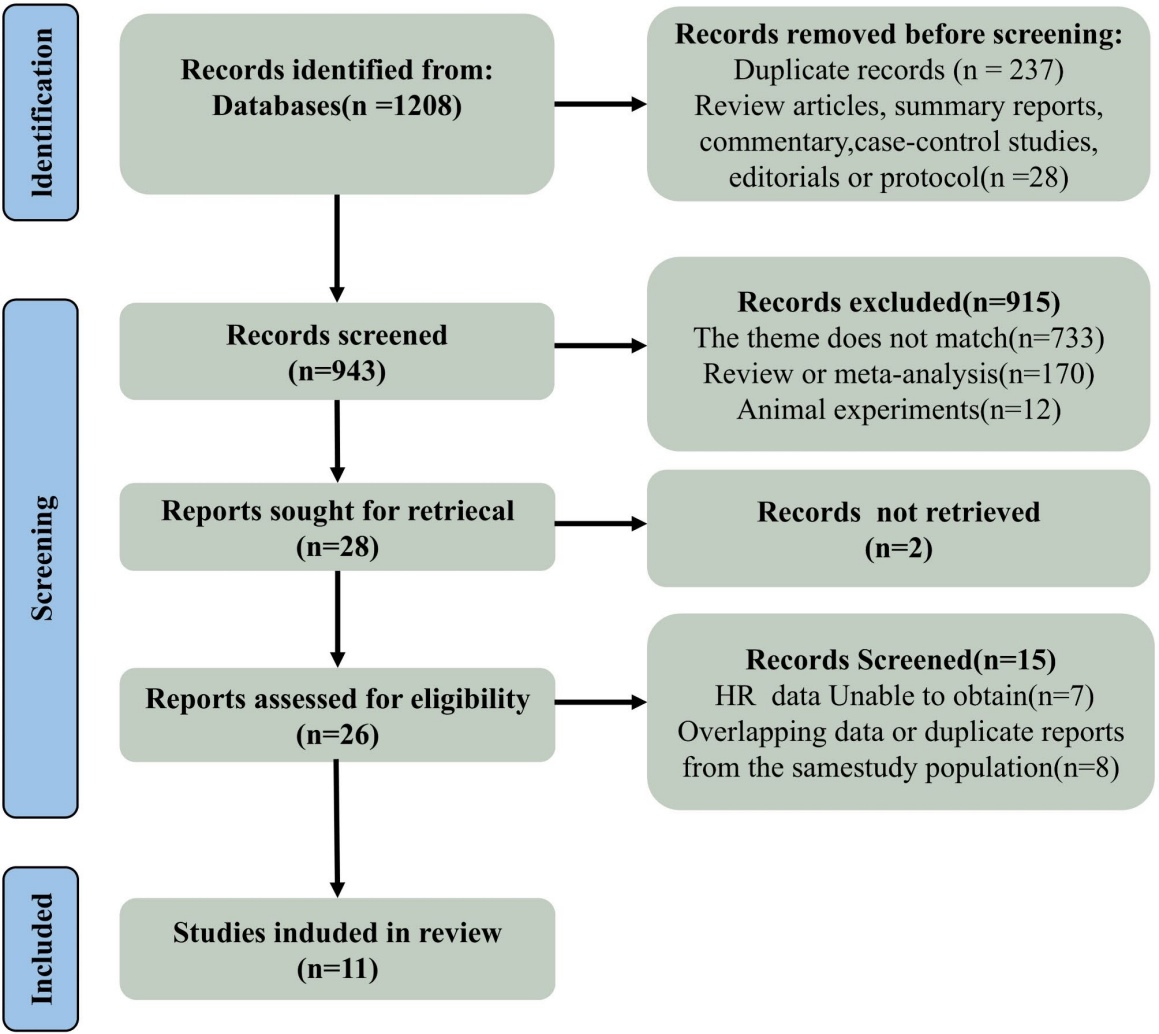
PRISMA flow chart. Abbreviation: HR, hazard ratio

A flowchart of the literature search, screening, and eligibility assessment process is shown in Fig. 1. We identified a total of 1208 potentially relevant studies, of which 237 were duplicates. We screened 971 records based on titles and abstracts, excluding 943 studies for the following reasons: irrelevant topics (*n* = 733), review articles, commentaries, or editorials (*n* = 28), systematic reviews or meta-analyses (*n* = 170), and animal studies (*n* = 12). Full texts of the remaining 28 studies were assessed, and 17 studies were excluded: full text not available (*n* = 2), data published in duplicate (*n* = 8), no report or inability to extract HR from figures, or lack of univariate HR results (*n* = 7). Finally, 11 studies were included in the analysis [26, 27, 35–43].

### Characteristics of the included studies

Tables 1 and 2 provide detailed descriptions of the characteristics of the studies included in the meta-analysis and the relevant HRV parameters. We included a total of 11 cohort studies published between 2013 and 2023, comprising 2 prospective cohort studies and 9 retrospective cohort studies, involving 2539 patients in total. The smallest cohort consisted of 39 participants, while the largest included 651 participants. The studies included cases of lung cancer, colorectal cancer, pancreatic cancer, liver cancer, and cervical cancer. Two studies measured EGC for more than 5 min, while only three studies had measurements shorter than 5 min.

We assessed publication bias in the included studies using the Newcastle-Ottawa Scale (NOS) and found that 2 studies scored 9 points, 5 studies scored 8 points, and 4 studies scored 7 points, indicating an overall high quality of the literature (Supplementary Fig. 1).

Qualitative symmetry was observed in the funnel plots shown in Supplementary Fig. 3, which indicated no significant publication bias.

**Table 1:** Characteristics of the studies included in the meta-analysis

Year	Author	Country	Design	No. of patients	Age (years)	Cancer type	Stage (%)	Median survival time of all patients	Median follow-up time of all patients
2013	Wang [35]	China	Prospective	40	61 (39–75)	Mixed	Metastatic (100%)	—	3.80 months
2016	De Couck [36]	Belgium	Prospective	272	60.0 ± 11.5	Pancreatic cancer (PC)	Metastatic (100%)	41 days	—
2015	Kim [37]	South Korea	Retrospective	103	63 (33–89)	NSCLC	Metastatic or recurrent (79.6%)	181 days (rang: 12–645)	—
2015	Guo [38]	USA	Retrospective	651	60.6 ± 15.8	Mixed (Solid tumor and Hemato logical)	Metastatic (54.4%)	88 weeks (SDNN < 70ms)	—
2020	Strous [27]	Norway	Retrospective	428	67 ± 10	Colorectal cancer	I-III (100%)	—	61 months [IQR: 43–89]
2021	Ciurea [39]	Romania	Retrospective	231	—	Hepatocellular carcinoma	—	—	—
2022	Cherifi [40]	France	Retrospective	202	65.7 ± 11.6	Ovarian cancer	Metastatic (93%)	38.6 months	31 months
2022	Li [41]	China	Retrospective	56	60.4 ± 9.0	NSCLC and SCLC	Metastatic (100%)	—	19.7 months (rang: 1.0-28.7)
2023	McGovern [26]	UK	Retrospective	439	—	Colorectal cancer	I-III (100%)	—	78 months
2023	Wu [42]	China	Retrospective	39	62.7 ± 8.1	ES-SCLC	Metastatic (100%)	—	42.2 months
2023	Wu [43]	China	Retrospective	78	62.0 ± 9.3	NSCLC	Metastatic (100%)	—	21.4 months

Abbreviation: ES-SCLC: Extensive-Stage small cell lung cancer. NSCLC: Non-small-cell lung cancer

**Table 2:** HRV-related parameters of the studies included in this meta-analysis

Year	Author	HRV parameter	Cut off value of SDNN	Exclude cardiac disease	Duration of ECG recording	HRV analyzing system
2013	Wang [35]	①	10 ms	Yes	5 min	My ECGE3–80 portable ECG software (MSI, New Taipei City, Taiwan)
2016	De Couck [36]	①	20 ms	Yes	20s	The MUSE^®^ cardiology information system in the UZ Brussels hospital
2015	Kim [37]	①	20 ms	Yes	5 min	HRV analyzer-SA-3000P (Medi-Core Co. Ltd., Seoul, Korea)
2015	Guo [38]	①②	70 ms	Yes	20–24 h	Vision Premier 5 (Cardiac Science Corporation, Bothell, WA)
2020	Strous [27]	①②	20 ms	Yes	10s	12-lead 10-second ECG (150 Hz)
2021	Ciurea [39]	①②③④ ⑤⑥⑦⑧	110 ms	Yes	20–30 h	TLC5000 Holter ECG (Contec Medical Systems, Qinhuangdao, Hebei Province, China)
2022	Cherifi [40]	①	10 ms	Yes	10s	analyzed and interpreted by a confirmed cardiologist
2022	Li [41]	⑩⑪⑫⑬⑭	—	Yes	5 min	Kubios HRV Premium software (version 3.1.0, Kubios Oy, Kuopio, Finland)
2023	McGovern [26]	①②	24 ms	Yes	5 min	12-lead, 10s (150 Hz) pre-operative ECG
2023	Wu [42]	①②	7.6 ms	Yes	5 min	Kubios HRV Premium software (version 3.1.0, Kubios Oy, Kuopio, Finland)
2023	Wu [43]	①② ⑦⑧⑨⑩ ⑪⑫⑬⑭	23.5 ms	Yes	5 min	Kubios HRV Premium software (version 3.1.0, Kubios Oy, Kuopio, Finland)

HRV Parameter: ①SDNN ②RMSSD ③MSD ④pNN50 ⑤ULF ⑥VLF ⑦LF ⑧HF ⑨LF/HF ⑩Lmax ⑪Lmean ⑫REC ⑬DET ⑭ShanEn

### Meta-analyses

#### 1. Time domain parameters

##### Higher SDNN VS. lower SDNN

**Fig. 2:**
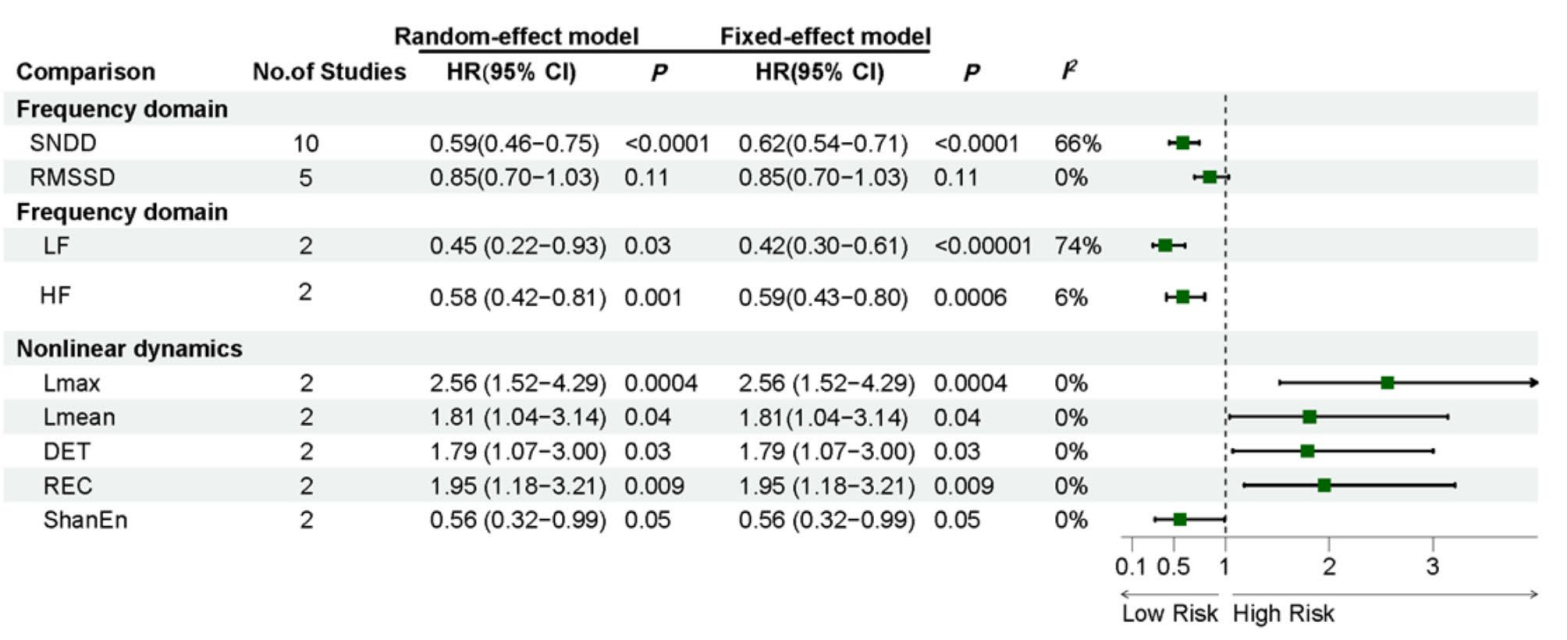
Effect of different HRV parameters on tumor prognosis. using HR for OS as raw data. Abbreviation: CI, confidence interval; HR, hazard ratio

In this analysis, we included 10 studies, with a total *I*^*2*^ of 66% and an HR of 0.59 (95% CI: 0.46–0.75; *P* < 0.0001), revealed that OS was significantly longer in the higher SDNN group than in the lower SDNN group (Fig. 2). To assess the impact of individual studies on the overall effect size, we conducted a sensitivity analysis by sequentially excluding each study and re-running the meta-analysis. The point estimates of the combined effect sizes consistently fell within the 95% CI of the overall effect size, indicating that the removal of any single study had minimal impact on the overall results, thereby demonstrating the stability of our study (Supplementary Fig. 2).

##### 1. Higher RMSSD VS. lower RMSSD

A total of 5 studies were included in this analysis, including Lung cancer (*n* = 2), colorectal cancer (*n* = 2), and hepatocellular carcinoma (*n* = 1), with a total *I*^*2*^ of 0% and an HR of 0.85 (95% CI: 0.70–1.03; *P* = 0.11), revealed that OS was non-significantly longer in the higher RMSSD group than in the lower RMSSD group (Fig. 2).

#### 2. Frequence domain parameters

##### Higher LF VS. lower LF

The results of the higher LF versus lower LF analysis are shown in Fig. 2, we included 2 studies, covering lung cancer and hepatocellular carcinoma. The results were statistically significant (*I²* = 74%, HR = 0.45, 95% CI: 0.22–0.93; *P* = 0.03).

##### Higher HF VS. lower HF

Two studies were included in the analysis of higher HF versus lower HF, with a total *I*^*2*^ of 6% and an HR of 0.59 (95% CI: 0.43–0.80; *P* = 0.006), and the results were statistically significant (Fig. 2).

#### 3. Nonlinear parameters

This analysis results are shown in Fig. 2, we included 2 studies. Since there was no heterogeneity between the studies, a fixed-effects model was used to combine the effect sizes. The combined effect estimates for L max, L mean, REC, DET and ShanEn were (HR = 2.56, 95% CI: 0.52–4.29; *P* = 0.0004), (HR = 1.81, 95% CI: 1.04–3.14; *P* = 0.04), (HR = 1.79, 95% CI: 1.07-3.00; *P* = 0.03), (HR = 1.95, 95% CI: 1.18–3.21; *P* = 0.0009), and (HR = 0.56, 95% CI: 0.32–0.99; *P* = 0.05).

## Discussion

### Main finding

This meta-analysis focused on the relationship of different parameters of HRV and with the prognosis of patients with cancer. Our study suggests that among time-domain parameters, the relationship between SDNN and tumor OS varies by tumor type. For digestive system and other cancers, higher SDNN is a protective factor for OS. Notably, this association is not supported for respiratory system tumors. However, all three studies included in this research focused on metastatic lung cancer. SDNN appears to be more susceptible to respiratory influences, such as respiratory sinus arrhythmia (RSA) [44, 45]. The respiratory pressure in advanced lung cancer may introduce errors in SDNN measurements, thereby affecting its prognostic value. Furthermore, De Couck et al. indicated that SDNN significantly predicts survival time in cancer patients under 65 years old, independent of confounding factors, but this was not observed in patients over 65 [36]. This suggests that the prognostic value of SDNN may be modulated by age.

**Fig. 3:**
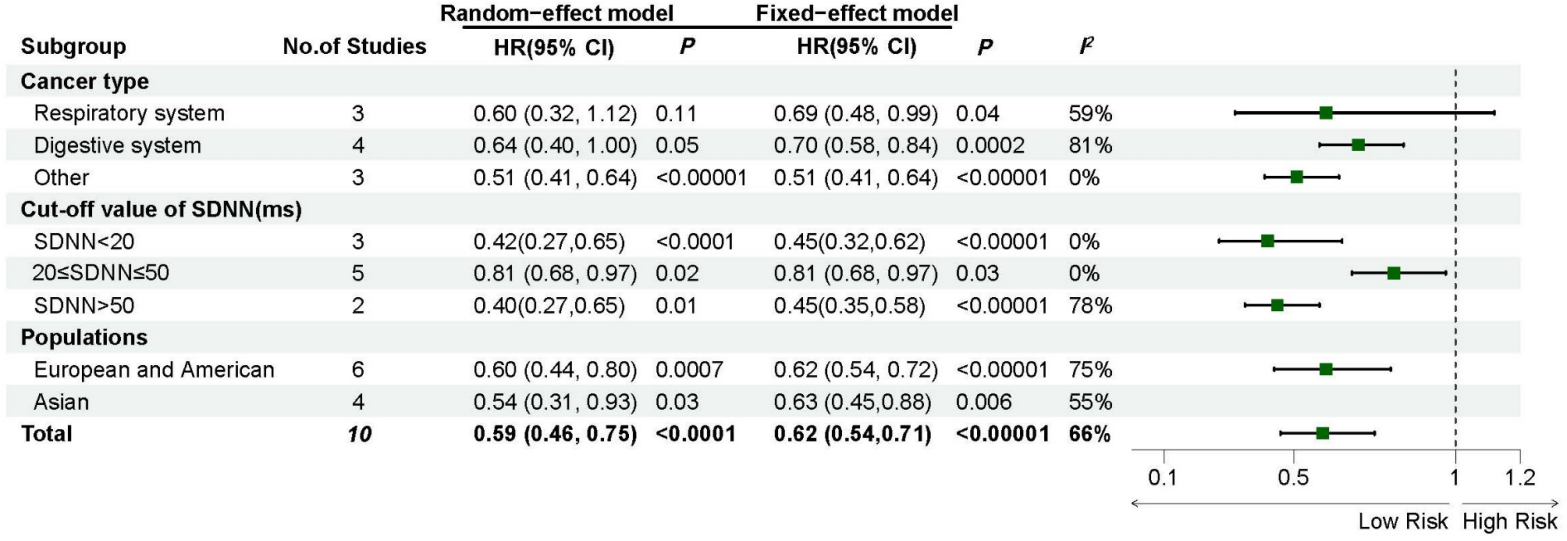
Subgroup analysis of the impact of higher SDNN and lower SDNN on tumor prognosis, using HR for OS as raw data. Abbreviations: CI, confidence interval; HR, hazard ratio

**Fig. 4:**
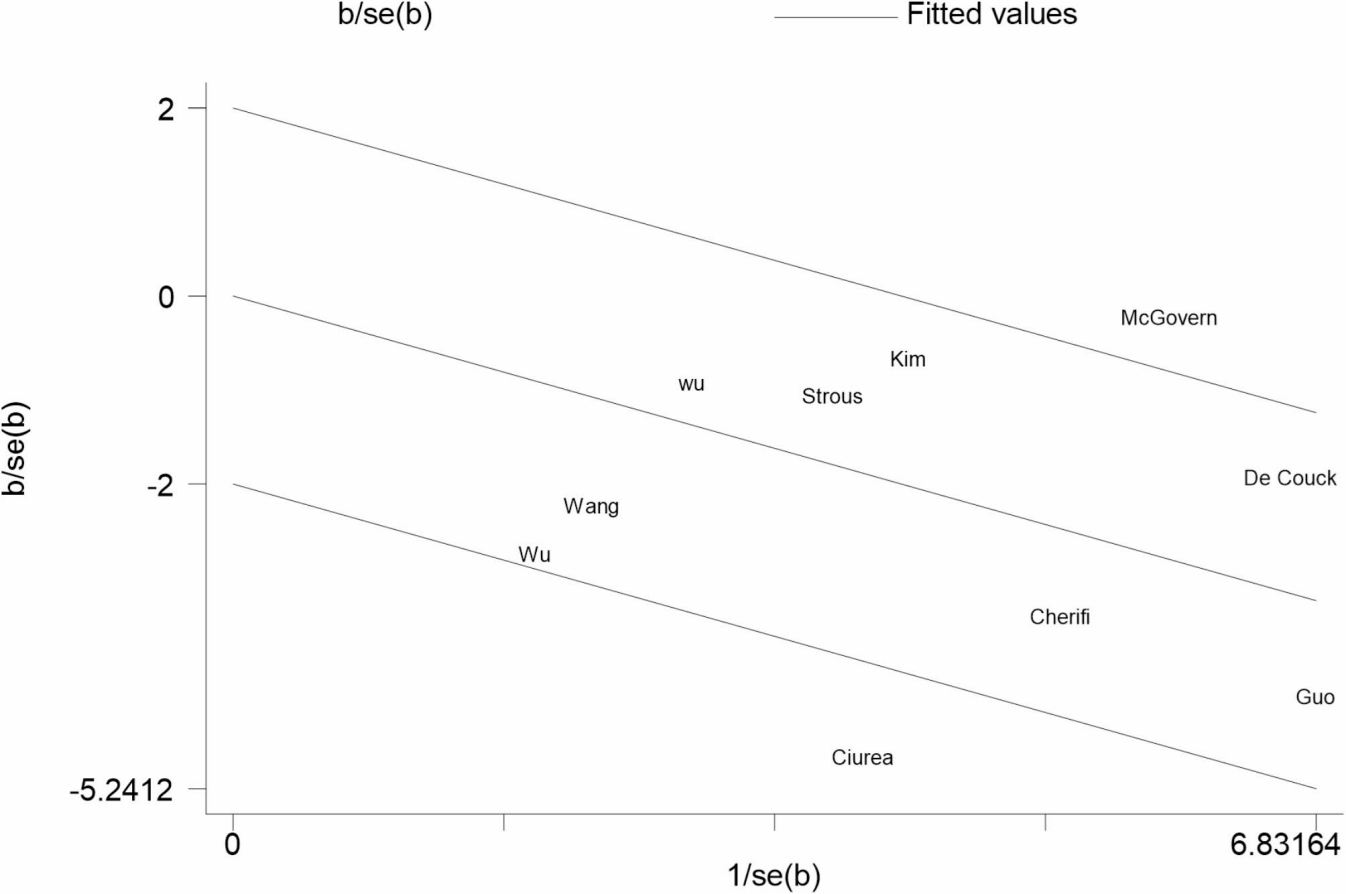
Galbraith radial plot of higher SDNN and lower SDNN on tumor prognosis

Failed to identify significant contributions of predefined covariates to the observed heterogeneity (*I²* = 66%) (Fig. 3). we constructed a Galbraith radial plot and identified two studies that fell outside the 95% confidence interval (Fig. 4). Notably, the cut-off value of SDNN (110 ms) in the study by Ciurea et al. was derived from comparisons with healthy controls and utilized 24-hour Holter monitoring for HRV assessment, differing from the 5-minute short-term ECG protocol recommended by the European Society of Cardiology [46]. McGovern et al. focused on patients with stage I-III colorectal cancer and found no association between time-domain parameters and tumor prognosis [26]. The majority of studies demonstrating a significant association between SDNN and cancer survival outcomes have predominantly involved patients with locally advanced or metastatic disease [35, 40, 47]. This discrepancy suggests that SDNN thresholds may serve as a potential confounding factor. Furthermore, this divergence may reflect underlying pathophysiological differences in autonomic regulation between localized and systemic cancers, emphasizing the need for stage-specific HRV interpretation frameworks.

RMSSD is the most specific estimator for the cardiac vagal tone due to its lower susceptibility to respiratory frequency influences [48]. Our results demonstrated RMSSD did not demonstrate statistically significant prognostic value for OS in cancer patients. (*P* = 0.11). However, Wu et al. identified through multivariate analysis that RMSSD showed significant correlation with OS in ES-SCLC patients after adjusting for KPS scores, suggesting potential masking effects of KPS on RMSSD’s true association [43]. Conversely, Strous et al. found that even after adjusting for potential confounding factors, there was no significant association between RMSSD and cancer prognosis, which may be attributed to the lower proportion of advanced stage tumors in their cohort [27]. In the future, the prognostic value of RMSSD needs to be validated under standardized conditions.

Higher HF in patients with recurrent or metastatic breast cancer is closely related to their longer overall survival [49]. Our meta-analysis further supports this. In contrast, while elevated LF showed a survival benefit, heterogeneity (*I²* = 74%) likely stems from differences in Cancer types, Wu et al. included advanced NSCLC [43], whereas Ciurea et al. analyzed HCC at diagnosis [39]. Similarly, Petrescu et al. reported that frequency domain indicators showed no significant association with median survival in pancreatic cancer (PC) patients, which may be attributed to the extensive sympathetic innervation of the pancreas [50, 51]. This suggests that the predictive value of frequency domain parameters may be influenced by tumor type.

Heart rate regulation exhibits inherent nonlinear complexity, nonlinear parameters can more effectively characterize the complexity of cardiac regulatory mechanisms in cancer patients [52]. Our analysis identified elevated Lmax, Lmean, REC, and DET as predictors of worse prognosis. Notably, Lmax remained an independent prognostic marker in lung cancer patients with brain metastasis even after multivariate adjustment [41]. These findings underscore the prognostic value of nonlinear HRV parameters in oncology, while necessitating further investigation to elucidate their precise pathophysiological mechanisms underlying cancer prognosis.

### Mechanisms

The predictive value of HRV for cancer Prognosis can be attributed to the “protective” role of VN. Accumulating evidence has demonstrated that an intact VN exerts protective effects against tumor progression (Table 3). It may regulate tumor progression through mechanisms such as suppressing inflammation, modulating immune and maintaining the balance between the sympathetic and parasympathetic.

**Table 3:** Evidence for a role of the vagus nerve in cancer development

Study	Year	Species	Vagal stimulation/ vagotomy	Cancer type	Results
Nelson [4]	1992	Rat	Vagotomy	Colorectal tumor (DMH)	Trend towards increased incidence and yield of colorectal and duodenal tumors
Mullan [5]	1990	Human	Undergone truncal vagotomy	Patients treated for peptic ulcers	Increased risk of colorectal tumors
Toftgaard [7]	1989	Human	Vagotomy	Patients treated for peptic ulcers	Increased risk of stomach cancer
Ahsberg [9]	2009	Human	Parietal cell vagotomy (PCV)	Patients treated for peptic ulcers	Increased risk of prostate carcinoma
Erin [10]	2008	Mice	Unilateral or bilateral vagotomy	Breast carcinoma (4THM cancer cell)	Increased lung, liver and kidney metastases
Erin [11]	2013	Mice	Right vagotomy or left vagotomy	Breast carcinoma (4THM cancer cell)	Increased intra- and extra-adrenal metastasis
Bernhard [12]	2018	Mice	Subdiaphragmatic vagotomy	Pancreatic ductal adenocarcinoma (PDAC)	Accelerates pancreatic tumorigenesis
Hiramoto [14]	2017	Mouse	Hepatic vagotomy	Liver metastasis of colorectal cancer	Exacerbation of liver metastasis and the survival rate
Partecke [60]	2017	Mice	Subdiaphragmatic vagotomy	Pancreatic cancer (6606 PDA)	Enhanced tumor growth, decreased survival time, suppressed TNFα production by TAMs
Sammi [6]	2018	Mistar albino rats	Pharmacological stimulation (Galantamine)	Colon cancer (DMH)	Decreased aberrant crypt focicount (ACF)
Erin [13]	2012	Mice	Pharmacological stimulation (Semapimod)	Breast carcinoma (4THM cancer cell)	Increased lung and liver metastases
Rawat [15]	2019	Wistar Rats	Transcutaneous auricular vague nerve stimulation(taVNS)	Colon cancer (DMH)	Counteract 1, 2-Dimethylhydrazine Induced Colon Carcinogenesis
Dubeykovskaya [17]	2016	Mice	Subdiaphramatic electrical stimulation	Colon cancer	Suppression of MDSC and cancer by vagally modulated TFF2 expression

1. **Suppress inflammation** Chronic inflammation and tumor progression go hand in hand. VN may suppress oxidative stress and “reflexively” mitigate inflammation through the cholinergic anti-inflammatory pathway (CAP), thereby potentially improving tumor prognosis [53, 54]. VN can locally exert anti-inflammatory effects by reducing the production of pro-inflammatory cytokines, such as tumor necrosis factor-alpha (TNF-α), through acetylcholine (ACh) and its receptor, α7 nicotinic acetylcholine receptor (α7nAChR) [55–57]. Additionally, the VN can modulate cardiac vagal tone and TNF-α [58, 59].
2. **Modulating immunity** The VN is an important conduit for bidirectional communication between the brain and the immune system, and may potentially regulate immune suppression to modulate tumor progression [60, 61]. Activation of the VN has been shown to inhibit the production of TNF-α [62, 63], whereas vagotomy leads to an increase in TNF-α levels in immune cells, such as macrophages and neutrophils. Antonica et al. demonstrated that sectioning the right VN reduces the number of lymphocytes released from the thymus, whereas electrical stimulation of the VN induces a temporary increase in their release through experiments on rats [65]. Additionally, the cholinergic system on immune cells is likely to play a role in regulating immune responses [65].
3. **Regulating the balance of the sympathetic and parasympathetic** VN and the sympathetic nervous system maintain a dynamic balance, working together to preserve homeostasis in the body [66, 67]. Excessive sympathetic nervous activity can promote angiogenesis through norepinephrine and accelerate tumor progression [68, 69]. Numerous studies have shown that VN stimulation can attenuate the sympathetic stress response and inhibit tumor growth, which may be associated with a reduction in plasma TNF-α levels [70–72].

Furthermore, VN can also influence tumor progression by modulating components of the tumor microenvironment, such as gut microbiota and stem cells [62, 73, 74]. However, the complexity of VN and its extensive branching make it challenging to determine its exact role in tumor progression. Reijmen et al. indicates that taVNS alone or in combination with radiotherapy fails to suppress tumor growth [75]. VN may promote the occurrence and metastasis of prostate cancer and colon cancer through acetylcholine (ACh) [76, 77]. Magnon et al. indicates that patients with tumors characterized by parasympathetic nerve fiber density exhibit a significantly increased propensity for biochemical recurrence and metastatic dissemination [78]. Therefore, some experts have suggested that the VN may slow down tumor development at the systemic level, while may promote tumor development at the local level, in advanced or metastatic cancer, VN activity may exhibit its protective effect [16, 79]. Further research is needed to explore the actual role and mechanisms of the VN in general cancer and its metastatic stage.

### Implications for future research

Future research should delve into the specific role of VNS in cancer therapy, including understanding the exact mechanism by which HRV changes are associated with cancer progression, effects on different tumor types and stages, thereby providing evidence-based support for the clinical application of VN modulation techniques in oncology. In clinical practice, it is recommended to incorporate HRV monitoring into routine cancer management, focus on the predictive value of nonlinear parameters for prognosis. Given the circadian rhythm of HRV (RMSSD and SDNN peak around 6 a.m.), it is advisable to standardize monitoring to the morning [80, 81]. HRV monitoring strategies should be dynamically adjusted based on cancer stage and treatment objectives, continuous monitoring during the treatment phase to detect early autonomic dysfunction, where reduced HRV may indicate chemotherapy-related cardiotoxicity risk in breast cancer patients [82]. Integrating HRV with multimodal data (e.g. CRP, IL-6, KPS) is recommended to enhance prognostic evaluation. Gidron et al. demonstrated that a new vagal neuroimmunomodulation (NIM) index, defined as the ratio of RMSSD to CRP, represents a novel independent prognostic biomarker for lethal cancers [83]. Moreover, Studies suggest that the LF/HF ratio may serve as a potential biomarker for psychological interventions targeting depressive states in cancer patients [84, 85]. Convenient and cost-effective monitoring can be achieved through 5-minute short-term HRV assessments combined with wearable devices (chest straps or wristbands), with HRV parameters seamlessly integrated into electronic medical record systems for real-time anomaly alerts (SDNN < 20ms).

### Strengths and limitations

Compared to prior meta-analyses [24], the present study demonstrates several methodological and conceptual advancements. Firstly, we expanded the sample size from 1,286 to 2,539 participants, enabling a comprehensive analysis of diverse HRV parameters (including time-domain, frequency-domain, and nonlinear indices), in relation to oncological outcomes. Systematic subgroup analyses were conducted according to tumor type, cut-off values of SDNN and populations to enhance clinical specificity. Secondly, this investigation was prospectively registered in the PROSPERO registry prior to data synthesis. To optimize measurement validity, stringent exclusion criteria were implemented to control for confounding factors affecting HRV assessments, particularly pre-existing cardiovascular comorbidities and pharmacotherapies with autonomic effects. Finally, we discussed the dualistic role of vagal nerve signaling in tumorigenesis, and discussed its implications for future research and clinical strategies. Unavoidably, this study still has some limitations. First, this is a meta-analysis based on cohort studies, and inherent bias may be present. Second, the included studies exhibit potential heterogeneity in terms of tumor types, stages, and SDNN cut-off values. The SDNN values are significantly influenced by ECG acquisition methods, as well as the duration and technique of HRV measurement. Third, due to the insufficient number of included studies, subgroup analyses for the frequency-domain and nonlinear HRV parameters were not conducted.

## Conclusion

The findings of this study demonstrate that HRV parameters, particularly SDNN, HF-HRV, and nonlinear parameters, exhibit predictive value for prognosis in cancer. Furthermore, it can be inferred that elevated VN activity may predict prolonged survival outcomes. However, these findings should be interpreted with caution due to the heterogeneity observed across included studies. Future research should prioritize prospective studies with standardized measurement protocols to validate these associations.

## Electronic supplementary material

Below is the link to the electronic supplementary material.

Supplementary Material 1

Supplementary Material 2

## Data Availability

All the research data and materials used in this study were obtained from pub-licly available sources. The following sources were used: PubMed, EMBASE, the Cochrane Library, and Web of Science.

## Abbreviations

HRV: Heart rate variability
VN: Vagus nerve
SDNN: Standard deviation of all normal-to-normal RR intervals
RMSSD: Root mean square standard deviations of RR intervals
HF-HRV: High-frequency power HRV
LF-HRV: Low-frequency power HRV
OS: Overall survival
Lmax: Maximal diagonal line length
Lmean: Mean diagonal line length
REC: Percent of recurrence
DET: Determinism
ShanEn: Shannon entropy
NOS: Newcastle-Ottawa Scale
HR: Hazard ratio
CI: Confidence interval
RSA: Respiratory sinus arrhythmia
ACh: Acetylcholine
CAP: Cholinergic anti-inflammatory pathway
TNF-α: Tumor necrosis factor-alpha
VNS: Vagus nerve stimulation
taVNS: Transcutaneous auricular vagus nerve stimulation
NIM: Neuroimmunomodulation
