# Supplementary material for: Vagal nerve activity and cancer prognosis: a systematic review and meta-analysis
Source: BMC Cancer. 2025 Mar 31;25:579. doi: 10.1186/s12885-025-13956-w (PMC11960028; doi:10.1186/s12885-025-13956-w)
Supplement: Supplementary file 1 — Supplementary Material 1 [file 12885_2025_13956_MOESM1_ESM.docx]

**Supplementary Appendix**

**Table 1.** The Population, Intervention, Comparator, Outcome, and Study design (PICOS) principles applied in this meta-analysis.

| **Terms** | **Definition** |
| --- | --- |
| **P**opulation | Patients who have been diagnosed with tumors |
| **E**xposure | Heart rate variability |
| **C**omparator | Cancer patients with higher and lower HRV were compared |
| **O**utcome | Overall survival(OS) |
| **S**tudy design | Retrospective or prospective cohort |

**Table 2.** Search strategy

| **Database** | **Search strategy** |
| --- | --- |
| **PubMed** | ((((prognosis[Title/Abstract]) OR (outcome[Title/Abstract])) OR (survival[Title/Abstract])) AND (((((neoplasm[Title/Abstract]) OR (carcinoma[Title/Abstract])) OR (cancer[Title/Abstract])) OR (adenocarcinoma[Title/Abstract]))) AND ((((heart rate variability[Title/Abstract]) ) OR (vagal nerve activity[Title/Abstract])) OR (HRV[Title/Abstract])) |
| **Web of Science** | (TI=( heart rate variability)OR TI=(HRV)AND(TI=(carcinoma)OR TI=(cancer))AND(TI=( prognosis)) OR TI=( outcome)) |

**Supplementary Figure 1.** Heatmap of the result of the quality of the included studies assessed by the Newcastle‒Ottawa Scale.


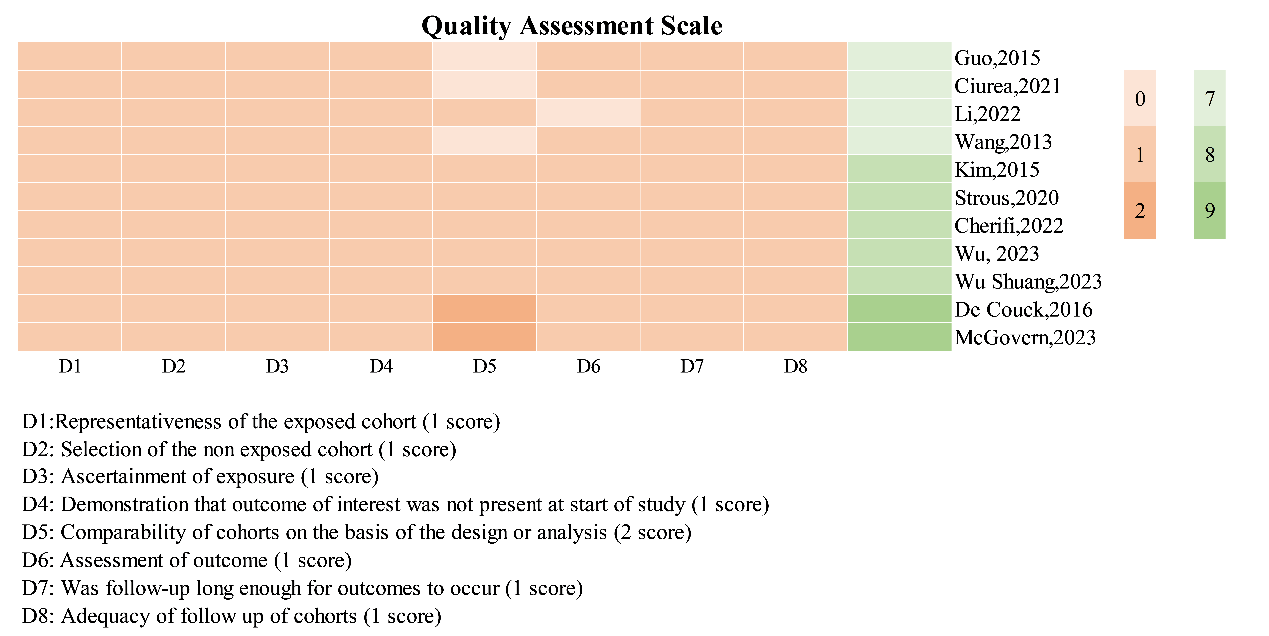


**Supplementary Figure 2.** Sensitivity analysis for higher SDNN VS. lower SDNN


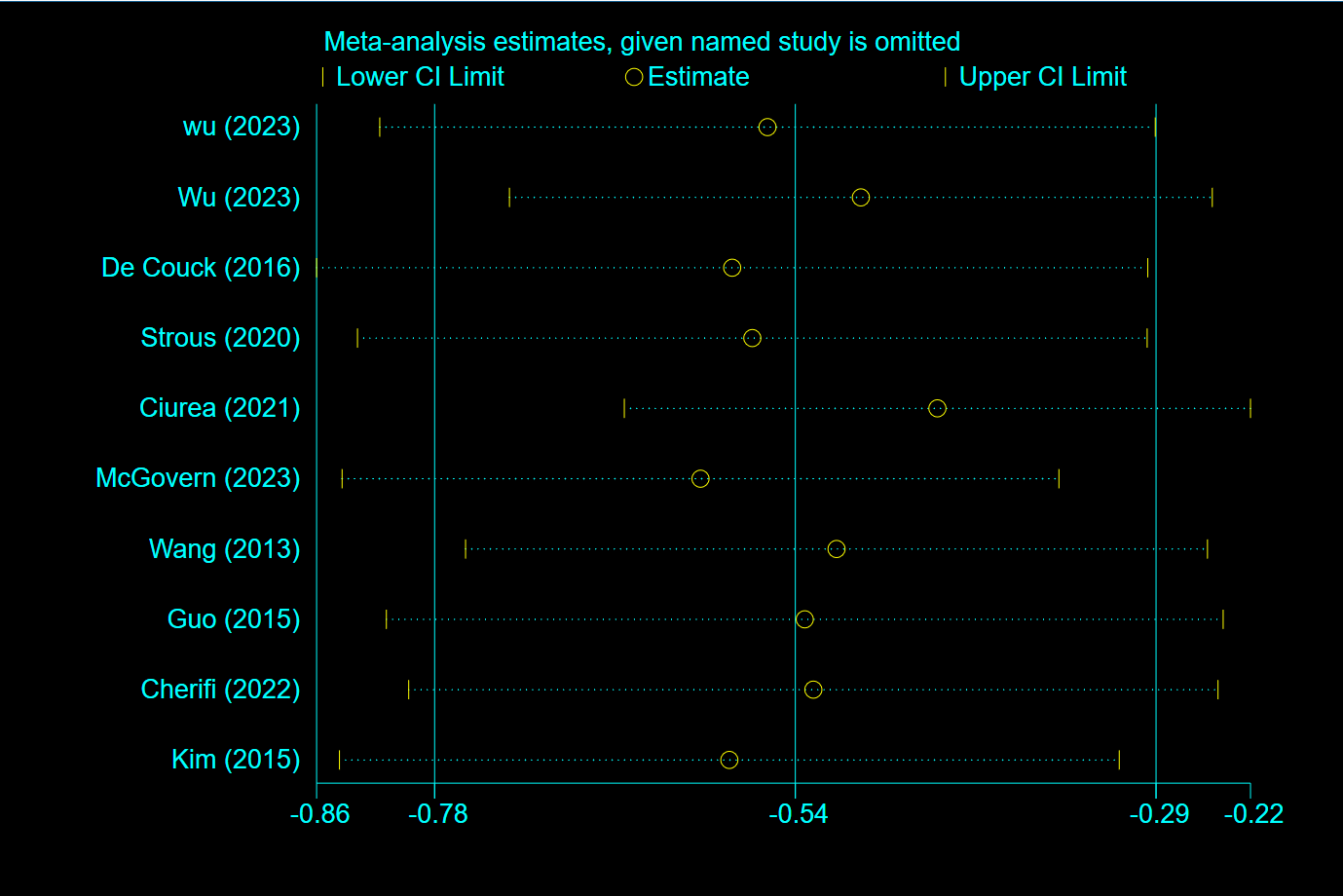


**Supplementary Figure 3.** Funnel Plots for each study.


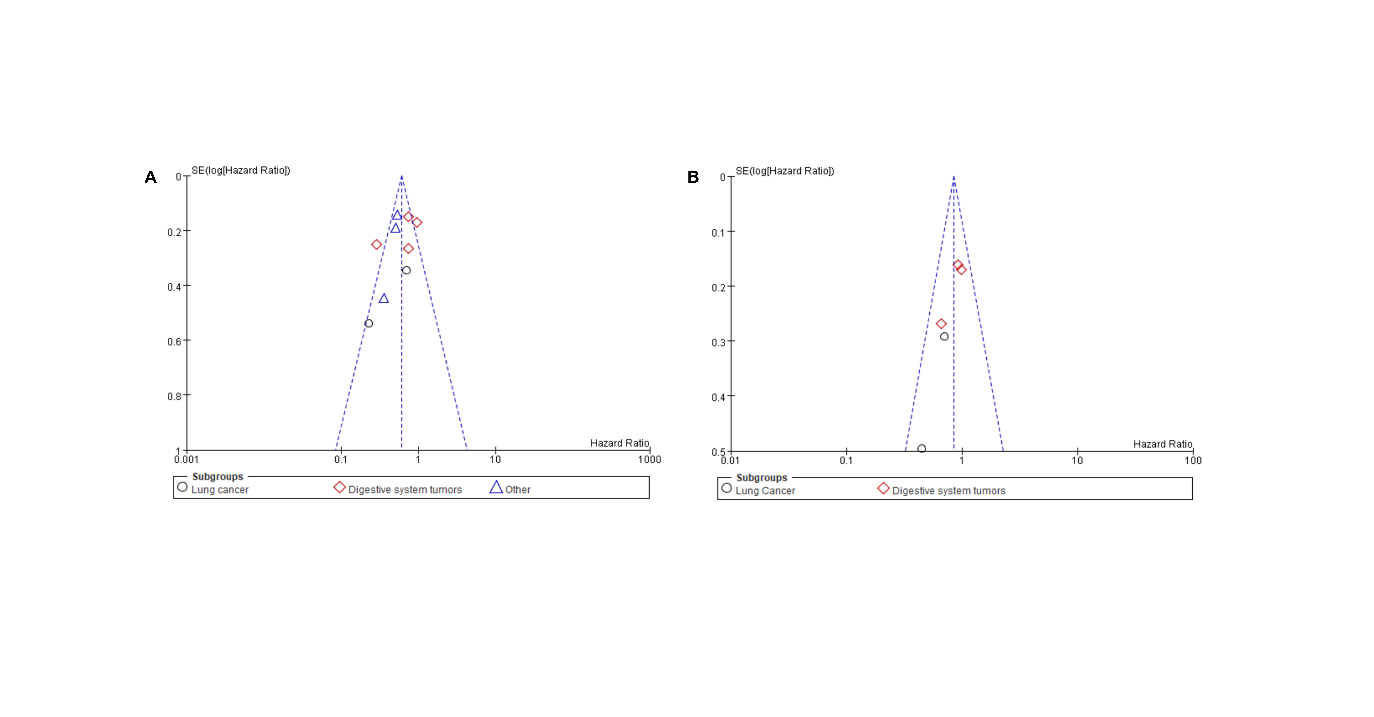


(A) **higher SDNN VS. lower SDNN** using HR for OS for tumours

(B) **higher RMSSD VS. lower RMSSD** using HR for OS for tumours
